# Optimizing Rituximab Maintenance Therapy: Outcomes of Extended-Interval Dosing in Multiple Sclerosis and Neuromyelitis Optica Spectrum Disorder

**DOI:** 10.14740/jocmr6529

**Published:** 2026-05-31

**Authors:** Supawit Kittipadakul, Tatchaporn Ongphichetmetha, Sasitorn Siritho, Ekdanai Uawithya, Naraporn Prayoonwiwat, Natthapon Rattanathamsakul, Jiraporn Jitprapaikulsan

**Affiliations:** a Department of Physiology, Faculty of Medicine Siriraj Hospital, Mahidol University, Bangkok 10700, Thailand; b Siriraj Neuroimmunology Center, Division of Neurology, Department of Medicine, Faculty of Medicine Siriraj Hospital, Mahidol University, Bangkok 10700, Thailand; c Division of Clinical Epidemiology, Department of Research and Development, Faculty of Medicine Siriraj Hospital, Mahidol University, Bangkok 10700, Thailand; d Bumrungrad International Hospital, Bangkok 10110, Thailand; e Division of Neurology, Department of Medicine, Faculty of Medicine Siriraj Hospital, Mahidol University, Bangkok 10700, Thailand

**Keywords:** Anti-CD20, B-cell depleting therapy, Multiple sclerosis, Neuromyelitis optica spectrum disorder, Rituximab

## Abstract

**Background:**

In many low-resource settings, access to several therapies for multiple sclerosis (MS) and neuromyelitis optica spectrum disorder (NMOSD) is still limited. Although rituximab (RTX) is considered off-label for some indications, it provides a relatively affordable alternative. However, its long-term use raises concerns about adverse effects such as secondary hypogammaglobulinemia and infection. Extending the dosing interval according to circulating cluster of differentiation 19 (CD19) B-cell counts has been proposed to preserve efficacy while minimizing complication and cost.

**Methods:**

This study retrospectively assessed the long-term efficacy and safety of RTX with CD19-guided, extended dosing intervals in patients with MS and aquaporin-4 immunoglobulin G-positive NMOSD. All treatments were delivered at Siriraj Hospital, Thailand, between January 1994 and February 2025. Clinical data, CD19 lymphocyte profiles, immunoglobulin levels and imaging findings were extracted for patients who had received RTX for at least 2 years.

**Results:**

Eighty-seven patients satisfied the inclusion criteria. In the MS cohort of 43 patients, treatment duration (mean ± standard deviation (SD)) was 4.10 ± 1.42 years, and the mean dosing interval was 32.61 ± 4.87 weeks. In 44 NMOSD patients, corresponding values were 4.92 ± 2.32 years and 33.87 ± 8.67 weeks. RTX reduced the median annualized relapse rate from 0.55 to 0.00 in MS and from 1.15 to 0.00 in NMOSD (both P < 0.001). The median Expanded Disability Status Scale scores improved from 2.0 to 0.0 in MS (P = 0.006) and from 4.5 to 4.0 in NMOSD (P < 0.001). Four patients maintained dosing intervals exceeding 48 weeks without relapse, and their CD19-positive B-cell proportion remained below 1%. Adverse events occurred in 32.6% of MS patients and 43.2% of NMOSD patients, most commonly infusion reactions (16.3% and 15.9%, respectively) or infections (14.0% and 27.3%, respectively). Leukopenia was documented in 4.7% of MS and 6.8% of NMOSD patients, whereas hypogammaglobulinemia arose only in NMOSD (6.7%); no fatal events were recorded.

**Conclusions:**

CD19-guided, extended-interval RTX is associated with relapse control, disability score improvement and favorable tolerability, while potentially lowering infusion frequency and healthcare costs in resource-constrained settings. Nonetheless, repopulation of CD19-positive B cells during prolonged intervals warrants vigilance because it may signal an increased risk of relapse.

## Introduction

Multiple sclerosis (MS) and neuromyelitis optica spectrum disorder (NMOSD) are common inflammatory demyelinating disorders of the central nervous system that impose substantial disability and socioeconomic burden.

In MS, inflammation and myelin loss are driven by the concerted activation of autoreactive T and B lymphocytes [1]. Activated T cells secrete cytokines—including interferon-γ, interleukin-17 and granulocyte–macrophage colony-stimulating factor—that perpetuate tissue injury [2]. Concomitantly, clonally expanded B cells mature into antibody-secreting plasmablasts, leading to excessive intrathecal immunoglobulin synthesis [3].

Pathogenesis in NMOSD is dominated by plasma cells that release aquaporin-4 immunoglobulin G (AQP4-IgG), the principal pathogenic antibody. Approved biological therapies for NMOSD now include inebilizumab, eculizumab, ravulizumab and satralizumab [4]. Nevertheless, in many low-resource settings, access to such agents remains restricted.

Rituximab (RTX), although still off-label for some indications, is considered relatively efficacious among other affordable alternatives [5–7]. Its long-term use, however, raises concerns about secondary hypogammaglobulinemia and infection, mandating a careful risk–benefit assessment [8, 9]. Individualizing treatment by extending the dosing interval according to circulating cluster of differentiation 19 (CD19) B-cell counts has been proposed to preserve efficacy while mitigating cumulative toxicity and cost [5, 10].

With standard 6-month dosing, RTX has shown to be efficacious in annualized relapse rate (ARR) and Expanded Disability Status Scale (EDSS) score in both MS and NMOSD, without serious adverse reactions. Comparable efficacy was also found for extended dosing regimen. Nevertheless, evidence regarding the long-term use of this regimen is still limited. Moreover, the optimal duration of interval prolongation to balance side effect mitigation relapse prevention remains uncertain. This study therefore examined the long-term efficacy and safety of CD19-guided, extended-interval RTX in MS and NMOSD [7, 8, 11, 12].

## Materials and Methods

### Study design

This retrospective cohort included patients with MS or AQP4-IgG–seropositive NMOSD treated at the Faculty of Medicine Siriraj Hospital—which comprises the university hospital (Siriraj) and the private Siriraj Piyamaharajkarun Hospital—between January 1994 and February 2025. The Siriraj Institutional Review Board approved the protocol (reference: Si 134/2025). Electronic medical records were reviewed to identify all patients with MS or NMOSD who had received RTX. This study was conducted in compliance with the ethical standards of the responsible institution on human subjects as well as with the Helsinki Declaration.

### Patients

Eligible patients had to satisfy three criteria. First, they were required to have MS diagnosed by the 2017 McDonald criteria [13] or AQP4-IgG–positive NMOSD diagnosed by the 2015 International Panel for NMO Diagnosis criteria [14]. Second, they had to receive RTX for more than 2 years. Third, dosing intervals had to exceed 24 weeks. Patients who defaulted from follow-up for ≥ 1 year after starting RTX were excluded.

Although the inclusion period dates back to 1994, all patients were longitudinally followed and underwent a formal audit to confirm that their diagnosis met the 2017 criteria for MS or the 2015 criteria for NMOSD. This process ensured that all historical cases were consistently reclassified according to contemporary diagnostic standards.

### Demographic and clinical data

The following variables were extracted from the electronic record: sex; age at diagnosis and at enrolment; date and indication for RTX initiation; and treatment status at that time. We also recorded the dosing interval and cumulative duration of RTX, as well as EDSS scores and neuroimaging obtained before and after treatment. Further variables included the time to first post-RTX relapse, serial CD19-positive B-cell percentages and serum immunoglobulin concentrations. Pre-RTX CD19 lymphocyte levels were collected before the initial dose, whereas post-RTX levels represent the average of all pre-infusion measurements taken from the second cycle through the last cycle.

### RTX dosing and infusion regimen

Following induction of RTX 1,000 mg on day 1 and day 15, maintenance infusions were conditionally scheduled every 6 months. The interval was tailored to CD19-positive B-cell repopulation. Infusions were deferred when the CD19-positive B-cell percentage was < 1%. CD19 counts were rechecked every 1–2 months, and RTX was administered once CD19 was > 1% or displayed a sustained upward trend toward that level. In patients with persistent CD19 suppression after several years of therapy, the maintenance dose could be reduced to 500 mg at the discretion of the treating neurologist.

### Outcome measurements

The primary objective was to evaluate the efficacy and safety of extended-interval RTX in patients treated for more than 2 years. Efficacy endpoints comprised time to first relapse, ARR and total relapse count. Disability and radiological activity were assessed using EDSS score and the appearance of new T2-weighted hyperintensity (new T2w) or new gadolinium-enhanced (new Gd) lesions on magnetic resonance imaging (MRI). ARR was calculated by dividing the cumulative number of relapses by the observation period, defined either from disease onset to RTX initiation or from RTX initiation to censoring. Safety was summarized as the number of patients experiencing any adverse event, infusion-related reaction, infection, leukopenia and hypogammaglobulinemia. Hypogammaglobulinemia was defined as the total serum immunoglobulin G concentration below the normal limit (below 548 mg/dL, according to the institution’s laboratory) [9]. The definition of progression independent of relapse activity (PIRA) in this study was mainly based on EDSS score. It was defined as a clinically significant increase in EDSS score from a reference score assessed more than 30 days before or 90 days after a relapse. A clinically significant increase was defined as an increase of 1.5 points from an EDSS of 0, 1.0 point from an EDSS of 1.0 to 5.0, or 0.5 point from an EDSS of 5.5 or higher [15].

### Statistical analysis

Categorical variables were summarized as numbers (percentages). Continuous data with a Gaussian distribution were reported as mean ± standard deviation, whereas non-normal data were presented as median (interquartile range (IQR)).

Group differences between MS and AQP4-IgG-seropositive NMOSD were assessed with the independent *t*-test for age, RTX duration and dosing interval. Non-parametric variables were compared with the Mann–Whitney U test for ARR and EDSS. Categorical data were analyzed with the Pearson χ^2^ or Fisher exact test for sex, prior treatment status, imaging findings and adverse events.

Within-subject comparisons employed the paired *t*-test for ARR and CD19-positive B-cell percentage, and the Wilcoxon signed-rank test for pre- versus post-treatment EDSS. Relapse-free survival was depicted with Kaplan–Meier curves. Subgroup analyses stratified patients by treatment status (treatment-naive vs treatment-experienced).

All computations were performed with IBM SPSS Statistics, version 29 (IBM, Armonk, NY, USA).

## Results

**Figure 1:**
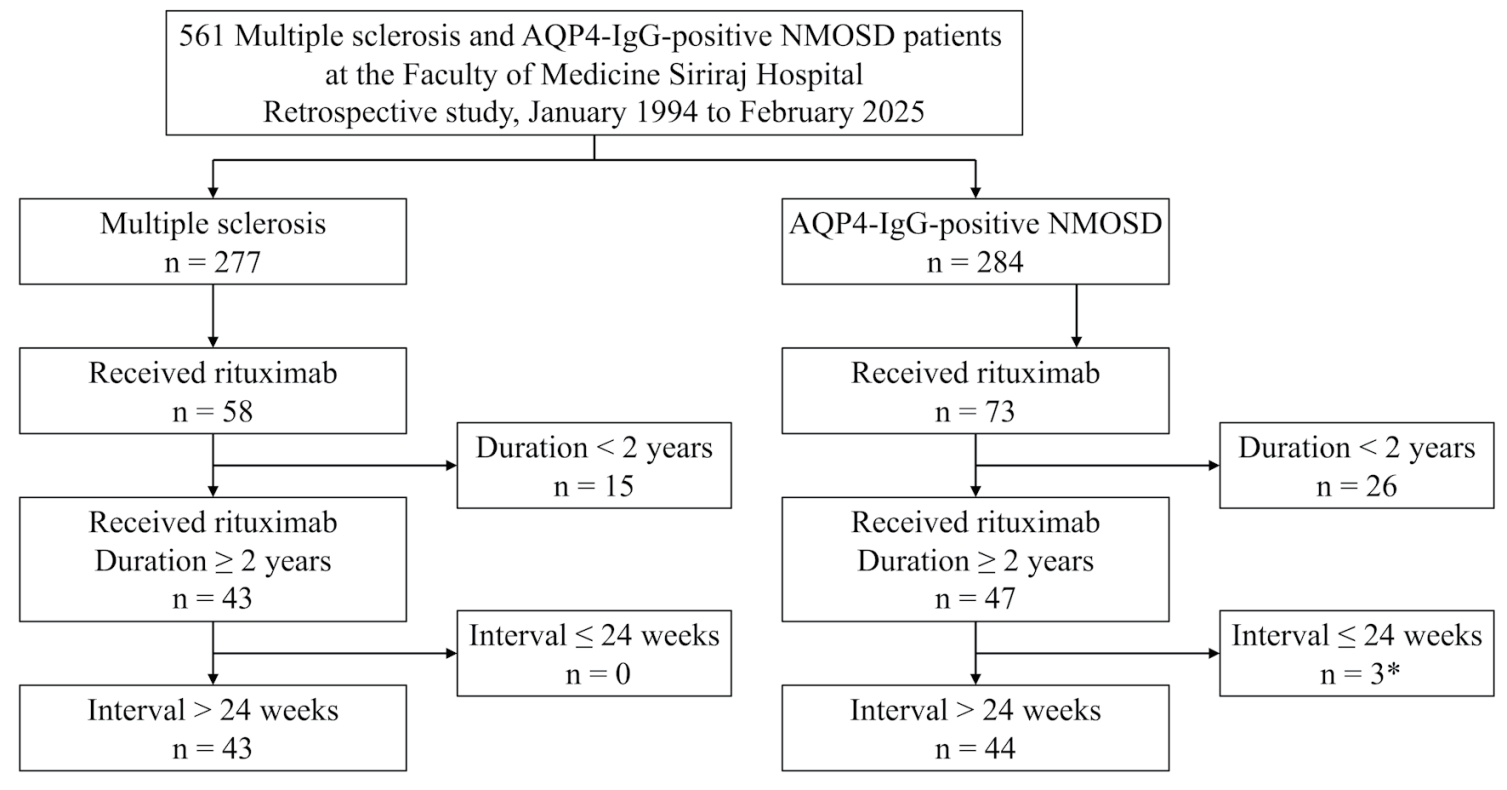
Flowchart demonstrating patients that were included and excluded in the study. *Three AQP4-IgG-seropositive NMOSD patients receiving rituximab were not recruited in the study because they had received rituximab at intervals shorter than 24 weeks due to persistently elevated CD19 levels 24 weeks after each infusion. AQP4-IgG+ NMOSD: aquaporin-4 immunoglobulin G seropositive neuromyelitis optica spectrum disorder.

Eighty-seven patients were included: 43 with MS (49.4%) and 44 with AQP4-IgG-seropositive NMOSD (50.6%) (Fig. 1). Most patients were female—31 of 43 MS patients (72.1%) and 37 of 44 NMOSD patients (84.1%). The mean age at disease onset was 26.63 ± 10.48 years for MS, and 41.35 ± 16.33 years for NMOSD. At RTX initiation, patients with MS were younger than those with NMOSD (32.34 ± 10.98 vs 46.23 ± 15.59 years). The median interval from disease onset to first RTX treatment was 5.10 years (IQR 1.49–8.05) for MS and 2.18 years (IQR 0.62–7.70) for NMOSD. Total RTX exposure averaged 4.10 ± 1.42 years in MS and 4.92 ± 2.32 years in NMOSD. The mean dosing interval was similar between groups (32.61 ± 4.87 weeks for MS and 33.87 ± 8.67 weeks for NMOSD) (Table 1).

**Table 1:** Baseline Characteristics of Multiple Sclerosis and Neuromyelitis Optica Spectrum Disorder

	Total (n = 87)	MS (n = 43)	NMOSD (n = 44)
Female sex	68 (78.2)	31 (72.1)	37 (84.1)
Age at onset (years)^a^	33.99 ± 15.52	26.63 ± 10.48	41.35 ± 16.33
Age at RTX initiation (years)^a^	39.28 ± 15.11	32.34 ± 10.98	46.23 ± 15.59
Age at enrolment (years)^a^	43.56 ± 15.47	36.28 ± 11.26	50.83 ± 15.79
Duration since onset to RTX initiation (years)^b^	3.28 (0.76–8.04)	5.10 (1.49–8.05)	2.18 (0.62–7.70)
Treatment status, n (%)			
Treatment-naive	27 (31.0)	13 (30.2)	14 (31.8)
Treatment-experienced	60 (69.0)	30 (69.8)	30 (68.2)
Azathioprine	50 (57.5)	23 (53.5)	27 (61.4)
Mycophenolate	29 (33.3)	11 (25.6)	18 (40.9)
Methotrexate	1 (1.1)	1 (2.3)	0 (0.0)
Mitoxantrone	1 (1.1)	0 (0.0)	1 (2.3)
Cyclophosphamide	1 (1.1)	0 (0.0)	1 (2.3)
Interferon-beta	3 (3.4)	2 (4.7)	1 (2.3)
Teriflunomide	2 (2.3)	2 (4.7)	0 (0.0)
Fingolimod	1 (1.1)	1 (2.3)	0 (0.0)
RTX indication, n (%)			
Treatment ineffectiveness	45 (75.0)	23 (76.7)	22 (73.3)
Treatment adverse effects	15 (25.0)	7 (23.3)	8 (26.7)
RTX regimen			
Initial dosing interval (weeks)^c^			
Mean ± SD	31.98 ± 8.01	30.98 ± 6.85	32.95 ± 8.97
Median (IQR)	27.71 (26.57–37.43)	27.71 (26.43–34.29)	28.00 (26.61–39.07)
Mean dosing interval (weeks)			
Mean ± SD	33.25 ± 7.04	32.61 ± 4.87	33.87 ± 8.67
Median (IQR)	30.38 (28.57–36.67)	32.25 (29.03–35.89)	29.44 (27.87–40.92)
RTX duration (years)			
Mean ± SD	4.52 ± 1.96	4.10 ± 1.42	4.92 ± 2.32
Median (IQR)	4.34 (3.10–5.51)	4.32 (2.81–5.46)	4.45 (3.16–5.52)

^a^Data are presented as mean ± standard deviation. ^b^Data are presented as median (IQR). ^c^Initial dosing interval refers to the dosing interval of the first cycle immediately after induction. NMOSD: neuromyelitis optica spectrum disorder; MS: multiple sclerosis; RTX: rituximab; SD: standard deviation; IQR: interquartile range.

RTX was the first treatment in 13 MS patients (30.2%) and 14 AQP4-IgG-seropositive NMOSD patients (31.8%). The remainder had previously received immunosuppression. Azathioprine had been prescribed to 23 MS patients (53.5%) and 27 NMOSD patients (61.4%), whereas mycophenolate mofetil was used in 11 MS patients (25.6%) and 18 NMOSD patients (40.9%). Among 30 treatment-experienced MS patients, 23 (76.7%) switched to RTX for lack of efficacy and seven (23.3%) because of adverse effects. In the 30 treatment-experienced NMOSD patients, 22 (73.3%) changed for ineffectiveness and eight (26.7%) because of adverse effects (Table 1).

### Efficacy in MS

Thirty-nine of 43 patients with MS (90.7%) remained relapse-free during follow-up, and the mean dosing interval was 32.61 ± 4.87 weeks. Relapse occurred in four individuals (Table 2). The first patient had relapsed 1.59 years after RTX initiation and 25 weeks after the last dose, with CD19 of 3.89% at relapse. The second patient had relapsed 2.16 years after RTX initiation and 24 weeks after the last dose, with CD19 of 2.91% at relapse. The third patient had relapsed 1.15 years after RTX initiation and 30 weeks after the last dose; CD19 data were unavailable. The last patient had relapsed 1.28 years after RTX initiation and 36 weeks after the last dose; CD19 data were also unavailable (Supplementary Material 1, jocmr.elmerjournals.com).

**Table 2:** Disease Activity and Disability Outcomes of Multiple Sclerosis and Neuromyelitis Optica Spectrum Disorder

Efficacy outcomes	Total (n = 87)	MS (n = 43)	NMOSD (n = 44)
Relapse			
Number of relapses	11 (12.6)	4 (9.3)	7 (15.9)
Relapses within 6 months after RTX, n (%)	4 (4.6)	0 (0)	4 (9.1)
Time to first relapse after RTX (years)^a^	1.12 (0.27–1.60)	1.44 (1.18–2.02)	0.45 (0.20–0.87)
EDSS			
EDSS before RTX^a^	4.0 (2.0–5.8)	2.0 (0.0–4.5)	4.5 (4.0–6.5)
EDSS after RTX^a^	2.0 (0.0–4.0)	0.0 (0.0–2.0)	4.0 (2.0–5.0)
EDSS change^a^	1.0 (0.0–2.0)	0.0 (0.0–2.0)	1.0 (0.0–2.9)
Radiological activity, n (%)			
New T2w lesions	0/40 (0.0)	0/26 (0.0)	0/14 (0.0)
New Gd lesions	0/40 (0.0)	0/26 (0.0)	0/14 (0.0)
Safety outcomes, n (%)			
RTX adverse effects	33 (37.9)	14 (32.6)	19 (43.2)
Infusion-related	14 (16.1)	7 (16.3)	7 (15.9)
Infection	18 (20.7)	6 (14.0)	12 (27.3)
Leukopenia	5 (5.7)	2 (4.7)	3 (6.8)
Hypogammaglobulinemia	1/27 (3.7)	0/12 (0.0)	1/15 (6.7)

^a^Data are presented as median (IQR). NMOSD: neuromyelitis optica spectrum disorder; EDSS: EDSS: Expanded Disability Status Scale; Gd: gadolinium-enhanced; MS: multiple sclerosis; RTX: rituximab; T2w: T2-weighted; IQR: interquartile range.

No patient received RTX more frequently than every 24 weeks.

**Figure 2:**
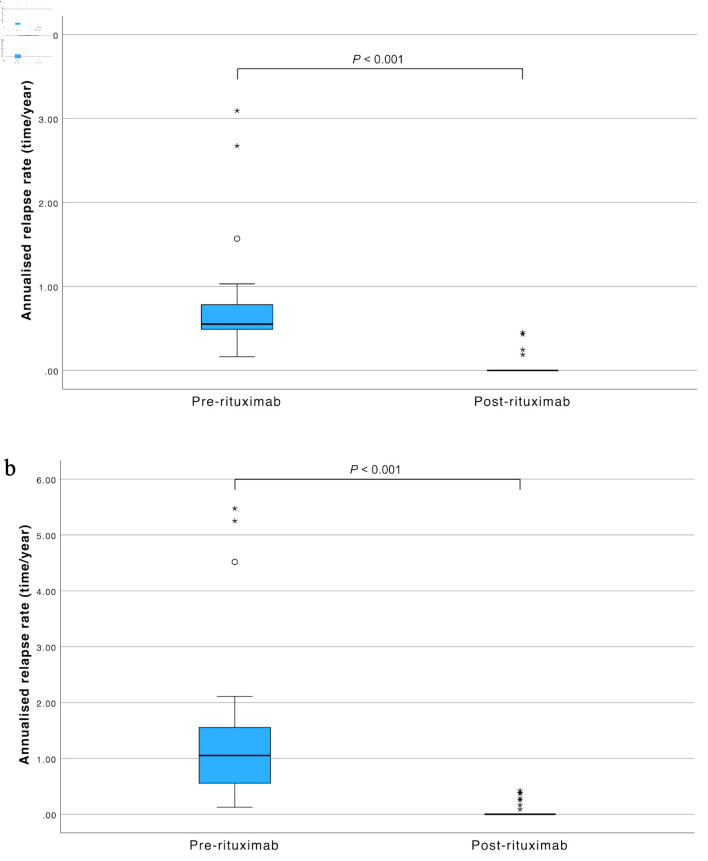
Annualized relapse rate pre- and post-treatment. (a) Multiple sclerosis. (b) AQP4-IgG-seropositive neuromyelitis optica spectrum disorder. AQP4-IgG: aquaporin-4 immunoglobulin G.

The median relapse count fell from three (IQR 2–4) before RTX to zero (IQR 0–0) afterwards (P < 0.001), and ARR decreased from 0.55 (IQR 0.47–0.84) to 0.00 (IQR 0.00–0.00) (Table 3, Fig. 2a).

**Table 3:** Comparison of Clinical and Laboratory Outcomes Between Pre- and Post-RTX

	Pre-RTX	Post-RTX	P value
MS (n = 43)			
Number of relapses (times)	3 (2–4)	0 (0–0)	< 0.001
ARR (times/year)	0.55 (0.47–0.84)	0.00 (0.00–0.00)	< 0.001
EDSS	2.0 (0.0–4.5)	0.0 (0.0–2.0)	0.006
%CD19	13.58 (9.11–18.25)	1.45 (0.95–2.56)	< 0.001
NMOSD (n = 44)			
Number of relapses (times)	3 (2–6)	0 (0–0)	< 0.001
ARR (times/year)	1.15 (0.54–1.72)	0.00 (0.00–0.00)	< 0.001
EDSS	4.5 (4.0–6.5)	4.0 (2.0–5.0)	< 0.001
%CD19	15.14 (12.25–23.38)	2.08 (0.89–5.39)	< 0.001

All data are presented as median (interquartile range). NMOSD: neuromyelitis optica spectrum disorder; ARR: annualized relapse rate; CD19: cluster of differentiation 19; EDSS: Expanded Disability Status Scale; MS: multiple sclerosis; RTX: rituximab.

In terms of disability progression, the median EDSS declined from 2.0 (IQR 0.0–4.5) pre-treatment to 0.0 (IQR 0.0–2.0) post-treatment (P = 0.006) (Table 3).

Among the 26 patients out of 43 patients who underwent MRI, no new T2w or new Gd lesions were detected during remission (Table 2).

### Efficacy in AQP4-IgG-seropositive NMOSD

Thirty-seven of 44 patients (84.1%) remained relapse-free during follow-up. The mean dosing interval was 33.87 ± 8.67 weeks. Seven patients (15.9%) relapsed, with a median time to first relapse of 0.45 years (IQR 0.20–0.87). Four of the seven relapses (57.1%) occurred within 6 months of RTX initiation; none of these four patients had received prednisolone (Table 2).

The median relapse count declined from three (IQR 2–6) before RTX to zero (IQR 0–0) afterwards, and ARR decreased from 1.15 (IQR 0.54–1.72) to 0.00 (IQR 0.00–0.00) (Table 3, Fig. 2b).

In terms of disability progression, the median EDSS improved from 4.5 (IQR 4.0–6.5) pre-treatment to 4.0 (IQR 2.0–5.0) post-treatment (P < 0.001) (Table 3).

Among 14 patients out of 44 patients who underwent imaging, no new T2w or new Gd lesions were detected during remission (Table 2).

CD19-positive B-cell percentages were significantly suppressed after RTX in both cohorts: from 13.58% to 1.45% in MS, and from 15.14% to 2.08% in NMOSD (both P < 0.001) (Table 3, Supplementary Material 2, jocmr.elmerjournals.com).

Four NMOSD patients (9%) received RTX at intervals exceeding 48 weeks; all had CD19 < 1% at infusion and remained relapse-free. Three MS and two NMOSD patients received a reduced 500-mg dose after a mean of 3.51 ± 1.33 years of therapy and stayed relapse-free for a further 2.38 ± 1.65 years.

**Figure 3:**
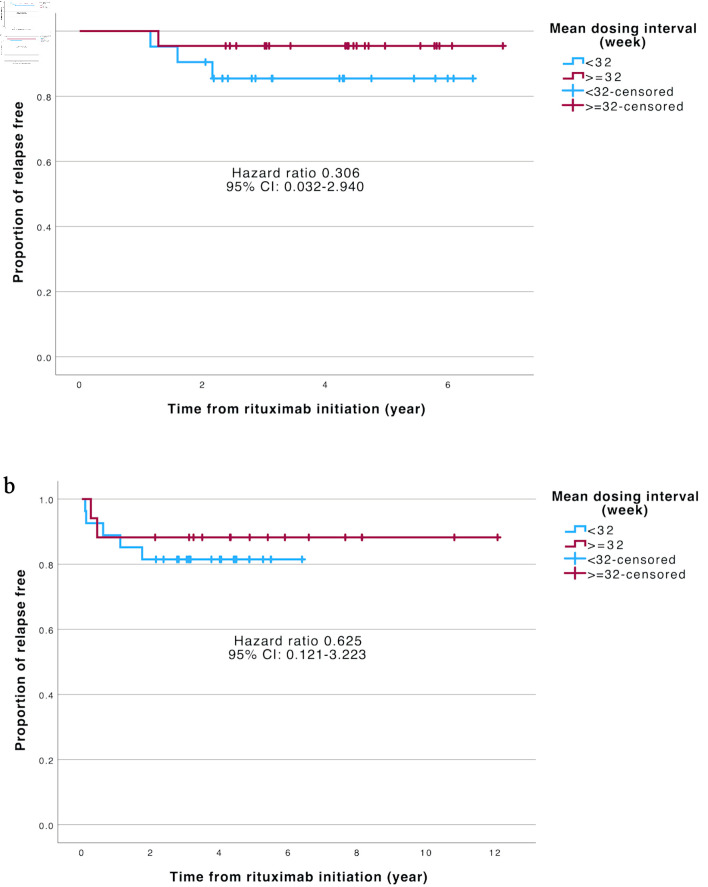
Kaplan–Meier curve demonstrating the proportion of relapse-free patients at the cutpoint of 32 weeks. (a) Multiple sclerosis. (b) AQP4-IgG-seropositive neuromyelitis optica spectrum disorder. CI: confidence interval; AQP4-IgG: aquaporin-4 immunoglobulin G.

Relapse-free survival did not differ between dosing intervals ≥ 32 weeks and < 32 weeks in either disease (Fig. 3).

### Safety

Adverse events occurred in 33 of 87 patients (37.9%): 14 of 43 with MS (32.6%) and 19 of 44 with AQP4-IgG-seropositive NMOSD (43.2%). Infusion-related reactions occurred in seven MS patients (16.3%) and in seven AQP4-IgG-seropositive NMOSD individuals (15.9%). Leukopenia developed in two patients with MS (4.7%) and three with NMOSD (6.8%). Hypogammaglobulinemia was reported in one NMOSD patient (6.7%). Infections were recorded in six MS patients (14.0%) and 12 NMOSD patients (27.3%) (Table 2). While most of the reported infections, including respiratory tract infection and urinary tract infection, were not serious, the NMOSD patient who had hypogammaglobulinemia experienced coronavirus disease 2019 (COVID-19) pneumonia with acute respiratory distress syndrome requiring mechanical ventilation (Supplementary Material 3, jocmr.elmerjournals.com).

### Subgroup analysis

**Figure 4:**
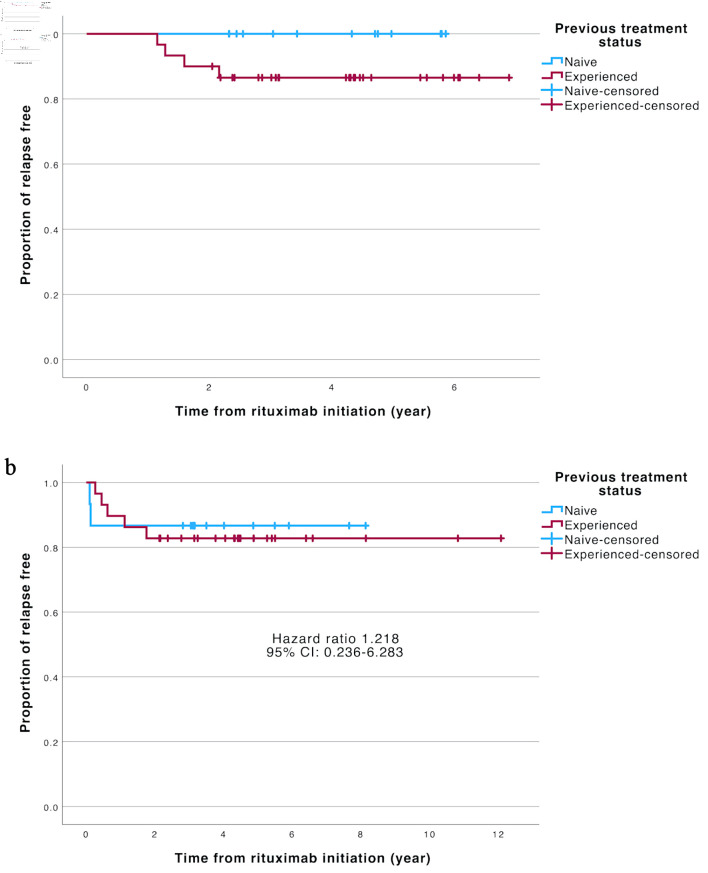
Kaplan–Meier curve demonstrating the proportion of relapse-free in treatment-naive and treatment-experienced patients. (a) Multiple sclerosis. (b) AQP4-IgG-seropositive neuromyelitis optica spectrum disorder. AQP4-IgG: aquaporin-4 immunoglobulin G.

Among patients with MS, the number of relapses significantly decreased in both the treatment-naive group (from 2 to 0; P = 0.001) and the treatment-experienced group (from 3 to 0; P < 0.001). EDSS scores improved only in the treatment-naive group (from 2.0 to 0.0; P = 0.012) (Table 4). In treatment-naive MS patients, hazard ratio (HR) could not be reliably calculated as there was no relapse event occurred in this group (Fig. 4a).

**Table 4:** Subgroup Analysis in Patients With and Without Other Treatments Prior to RTX

	Pre-RTX	Post-RTX	P value
MS			
Treatment-naive (n = 13)			
Number of relapses (time)	2 (1–3)	0 (0–0)	0.001
EDSS	2.0 (0.0–4.8)	0.0 (0.0–2.0)	0.012
Treatment-experienced (n = 27)			
Number of relapses (time)	3 (2–5)	0 (0–0)	< 0.001
EDSS	2.0 (0.0–4.0)	2.0 (0.0–3.0)	0.106
NMOSD			
Treatment-naive (n = 10)			
Number of relapses (time)	1 (1–2)	0 (0–0)	0.001
EDSS	4.5 (2.8–6.0)	2.0 (0.8–5.1)	0.011
Treatment-experienced (n = 24)			
Number of relapses (time)	4 (2–8)	0 (0–1)	< 0.001
EDSS	4.5 (4.0–6.5)	4.0 (2.1–4.0)	0.005

All data are presented as median (interquartile range). NMOSD: neuromyelitis optica spectrum disorder; EDSS: Expanded Disability Status Scale; MS: multiple sclerosis; RTX: rituximab.

In AQP4-IgG-seropositive NMOSD patients, a decrease in the number of relapses was also observed in both the treatment-naive (from 1 to 0; P = 0.001) and the treatment-experienced (from 4 to 0; P < 0.001) groups. EDSS scores significantly improved in both the treatment-naive (from 4.5 to 2.0; P = 0.011) and the treatment-experienced (from 4.5 to 4.0; P = 0.005) groups (Table 4). Relapse-free survival was comparable, with an HR of 1.218 (95% confidence interval (CI), 0.236–6.283) (Fig. 4b).

### PIRA

PIRA events occurred in two of 43 MS patients. The first patient started RTX 5.35 years after disease onset and continued treatment at 41.00-week intervals for 5.81 years. His EDSS score gradually increased from 0.0 to 6.0 over the course of treatment, despite no relapses. The second patient started RTX 5.52 years after onset and received it at 32.57-week intervals for 6.06 years. After experiencing five episodes of relapses, her EDSS score had reached 4.5 by the time of RTX initiation. Despite no further relapses after starting RTX, her EDSS score continued to progress to 6.0.

## Discussion

This retrospective cohort demonstrated that CD19-guided, extended-interval RTX was associated with marked relapse reduction and either stable or improved disability with acceptable tolerability in both MS and AQP4-IgG-seropositive NMOSD. Relapse incidence fell sharply, disability remained stable or improved, and some patients achieved annual infusion schedules. Reconstitution of CD19-positive B cells preceded several breakthrough relapses, underscoring the value of biomarker-guided dosing (Supplementary Material 1, jocmr.elmerjournals.com). It is noteworthy that RTX had been reported to be efficacious despite B-cell repopulation in some cases. While the data from this study are consistent with recent findings suggesting that not all repopulation leads to relapses, monitoring CD19 levels in both MS and NMOSD remains beneficial in many scenarios and may represent a safer practice to identify fast repopulators who may be at higher risk of relapses [10, 16].

Consistent with Spanish [17], Saudi Arabian [18], and Korean [19] cohorts, ARR fell to almost zero in both disorders. EDSS trajectories matched these data, either stabilizing or improving, again echoing prior MS [17, 18] and NMOSD [19] reports. Another recent comparative study between 6-month and 12-month dosing also found similar efficacy outcomes [10]. The double-blind RIN-I trial recorded a 37% relapse rate in AQP4-IgG-seropositive NMOSD treated with fixed-interval RTX [20, 21]. In contrast, the CD19/CD20/CD27-adjusted RIN-II study observed only two relapses among 33 patients (6.1%) during 20 months, with an ARR of 0.035 and a mean 9.5 ± 2.5-month infusion interval [20, 21]. Our CD19-guided, extended-interval regimen achieved comparable relapse suppression and disability outcomes, despite a follow-up that exceeded 4 years in many patients.

New T2w or Gd lesions on MRI presage clinical relapse [22, 23]. Previous studies showed that RTX suppresses such radiological activity [24, 25]. Our cohort likewise exhibited no new lesions throughout follow-up, supporting its sustained radiological efficacy. However, MRI was not obtained in all patients as a routine. Only a subset of this cohort (26/43 MS and 14/44 NMOSD) had imaging data available for review.

In this study, PIRA was observed in 4.65% of MS patients, which is consistent with previously reported findings ranging from 6 to 28% [26].

In Thailand, earlier work demonstrated short-term RTX efficacy with a median exposure of about 2.5 years [11]. We now extend these data, documenting durable benefit over roughly 4.5 years. Crucially, efficacy persisted although most patients received infusions at about 33 weeks—substantially longer than the conventional 24-week schedule—and without an upturn in relapse risk. This finding indicates that, in carefully selected patients, disease control can be maintained with less frequent dosing, conferring clear economic advantages in resource-limited settings.

Adverse events arose in 37.9% of patients, approximating the 44% previously reported in Thailand [11]. Leukopenia and hypogammaglobulinemia were uncommon. Infusion reactions occurred in 16.1% of patients and resolved either spontaneously or after slowing the infusion rate. Infections were more frequent in this series (20.7%) than in the earlier report (9.3%) [11], a disparity that likely reflects our longer observation period. The incidence of infections in this study was lower than most studies of standard dosing, which reported around 50% or more. Commonly reported infections were similar, being respiratory tract infection and urinary tract infection. Serious infections were similarly rare; however, they could typically be found in patients with hypogammaglobulinemia, ranging from 13 to 20%. Hypogammaglobulinemia were more prevalent in standard dosing regimen at around 17–22% [6, 8, 12, 19, 27–29].

Several limitations merit mention. First, the retrospective design resulted in missing data. Second, the power and generalizability of the findings was affected by a relatively small sample size despite recruiting every eligible patient at this large-scale institution, which reflects the limited accessibility of RTX until recently. Third, MRI was not obtained routinely, restricting the ability to confirm the absence of radiological activity in general population. Fourth, IgG concentrations and subclasses were not measured systematically prior to RTX initiation, limiting interpretation to post-treatment values in a small subset. Fifth, PIRA in MS patients in this study was assessed solely through changes of EDSS score, without including other composite measures such as upper limb function and cognition, which should be further explored in the future. Finally, the low number of post-RTX relapses curtailed the statistical power of subgroup analyses.

### Learning points

Short-term rituximab is an effective and generally safe treatment for MS and aquaporin-4-IgG-seropositive NMOSD. Lengthening the infusion interval has been proposed primarily as a strategy to mitigate long-term adverse effects, with the secondary benefit of reducing the overall financial burden.

Over a median 4.5-year follow-up, implementation of CD19-guided extended dosing protocol appeared to preserve near-complete relapse control and a favorable safety profile in both MS and NMOSD. CD19 monitoring helped schedule infusions, although isolated CD19 rebound did not always foreshadow clinical activity. In appropriately selected patients, intervals longer than 32 weeks were feasible without loss of efficacy, reducing drug use and clinic visits.

The results support rituximab as a long-term maintenance option in settings where licensed biologics are unavailable or unaffordable. They also justify further evaluation of personalized, CD19-guided, extended-interval dosing to limit adverse events and health-care expenditure while preserving therapeutic benefit.

## Supplementary Material

Suppl 1 Clinical data of patients with relapses.

Suppl 2 CD19 lymphocytes before each rituximab cycle.

Suppl 3 Infection details.

## Data Availability

The data that support the findings of this study are not openly available due to privacy reasons and are available from the corresponding author upon reasonable request.

## Generative AI and AI-assisted technologies in the writing process

During the preparation of this work the authors used ChatGPT in order to improve language and grammar. After using this tool/service, the authors reviewed and edited the content as needed and take full responsibility for the content of the published article.

## Abbreviations

AQP4-IgG: aquaporin-4 immunoglobulin G
ARR: annualized relapse rate
CD19: cluster of differentiation 19
EDSS: Expanded Disability Status Scale
Gd: gadolinium
IgG: immunoglobulin G
IQR: interquartile range
MRI: magnetic resonance imaging
MS: multiple sclerosis
NMOSD: neuromyelitis optica spectrum disorder
RTX: rituximab
T2w: T2-weighted
