# Supplementary material for: Optimizing Rituximab Maintenance Therapy: Outcomes of Extended-Interval Dosing in Multiple Sclerosis and Neuromyelitis Optica Spectrum Disorder
Source: J Clin Med Res. 2026 May 31;18(5):301–12. doi: 10.14740/jocmr6529 (PMC13278727; doi:10.14740/jocmr6529)
Supplement: Suppl 1 — Clinical data of patients with relapses. [file jocmr-18-05-301-s001.docx]

**Suppl 1.** Clinical data of patients with relapses

(A) Multiple sclerosis

| **Case** | **Time since RTX initiation to relapse (years)** | **Time since last RTX dose to relapse (weeks)** | **EDSS at relapse** | **CD19 during relapse (%)** |
| --- | --- | --- | --- | --- |
| 1 | 1.59 | 25 | 4.0 | 3.89 |
| 2 | 2.16 | 24 | 7.5 | 2.91 |
| 3 | 1.15 | 30 | N/A | N/A* |
| 4 | 1.28 | 36 | 2.5 | N/A* |

* Latest CD19 levels before relapse were 2.5% for patient 3 (28 days before, prompting re-administration upon follow-up visit) and 0.28% for patient 4 (37 days before).

(B) AQP4-IgG-seropositive neuromyelitis optica spectrum disorder

| **Case** | **Time since RTX initiation to relapse (years)** | **Time since last RTX dose to relapse (weeks)** | **EDSS at relapse** | **CD19 during relapse (%)** |
| --- | --- | --- | --- | --- |
| 1 | 0.27 | 14 | 7.5 | 0.31 |
| 2 | 1.75 | 12 | 4.0 | N/A* |
| 3 | 1.12 | 6 | 4.0 | 0.05† |
| 4 | 0.45 | 23 | 7.5 | 15.39 |
| 5 | 0.62 | 6 | 4.0 | 0.15† |
| 6 | 0.13 | 2 | 3.0 | N/A* |
| 7 | 0.10 | 5 | N/A | N/A* |

* Latest CD19 level before relapse was 1.67% for patient 2 (101 days before). No prior levels were available for patients 6 and 7.

† Patients 3 and 5 had relapses 45 and 22 days after latest RTX administration with pre-dosage CD19 levels of 3.11 and 6.33%, respectively.
