# Supplementary material for: Optimizing Rituximab Maintenance Therapy: Outcomes of Extended-Interval Dosing in Multiple Sclerosis and Neuromyelitis Optica Spectrum Disorder
Source: J Clin Med Res. 2026 May 31;18(5):301–12. doi: 10.14740/jocmr6529 (PMC13278727; doi:10.14740/jocmr6529)

**Suppl 2.** CD19 lymphocytes before each rituximab cycle

(A) Multiple sclerosis


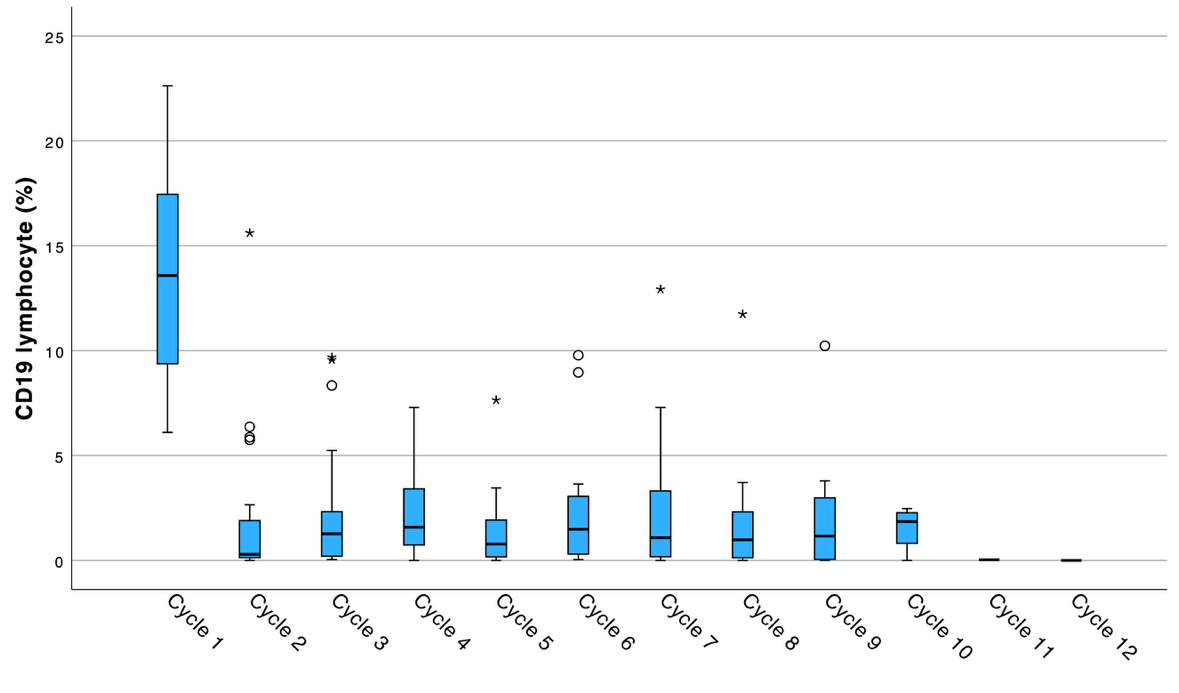


(B) AQP4-IgG-seropositive neuromyelitis optica spectrum disorder


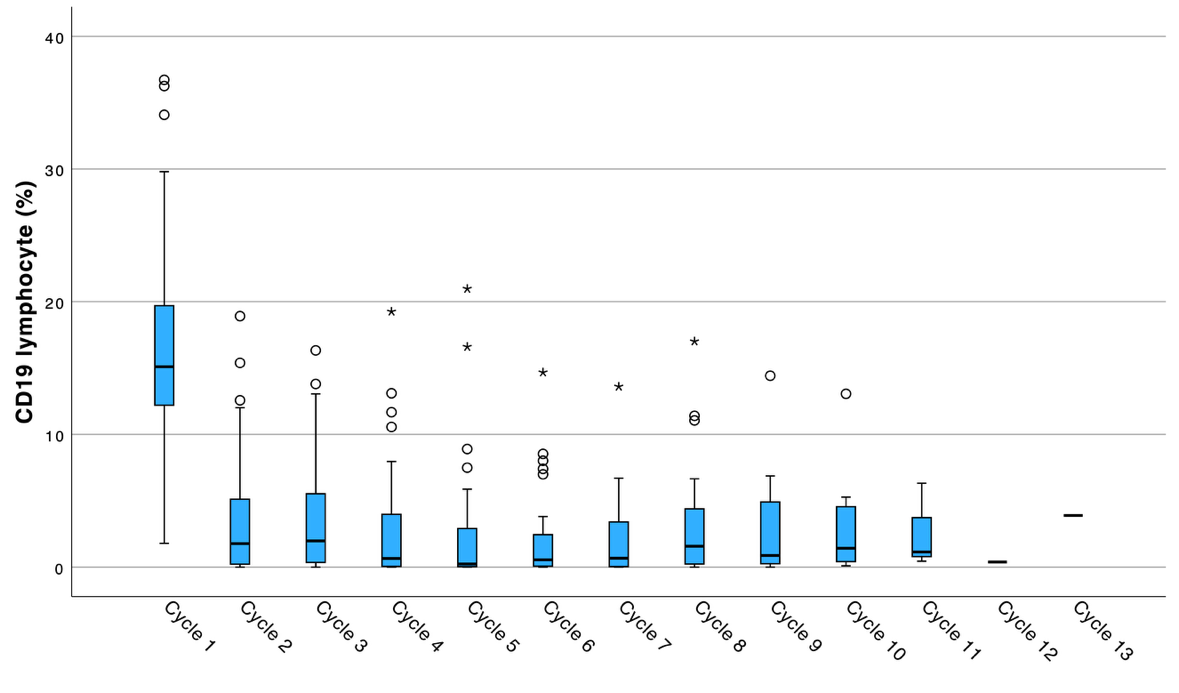

Supplement: Suppl 2 — CD19 lymphocytes before each rituximab cycle. [file jocmr-18-05-301-s002.docx]
