# Supplementary material for: Optimizing Rituximab Maintenance Therapy: Outcomes of Extended-Interval Dosing in Multiple Sclerosis and Neuromyelitis Optica Spectrum Disorder
Source: J Clin Med Res. 2026 May 31;18(5):301–12. doi: 10.14740/jocmr6529 (PMC13278727; doi:10.14740/jocmr6529)
Supplement: Suppl 3 — Infection details. [file jocmr-18-05-301-s003.docx]

**Suppl 3.** Infection details

(A) Multiple sclerosis

| **Case** | **Type of infection** | **Severity** | **Treatment** |
| --- | --- | --- | --- |
| 1 | Pneumonia | Non-severe | IV antibacterials for 3 days as OPD case |
| 2 | Urinary tract infection (*Proteus mirabilis*) | Non-severe | IV ceftriaxone as IPD case |
| 3 | Urinary tract infection (*Streptococcus agalactiae*) | Non-severe | IV ceftriaxone as IPD case |
| 4 | COVID-19 infection | Non-severe | PO molnupiravia for 5 days as OPD case |
|  | Labial abscess | Non-severe | Incision and drainage + PO co-amoxiclav for 5 days as OPD case |
| 5 | COVID-19 infection | Non-severe | N/A |
| 6 | COVID-19 infection | Non-severe | N/A |

(B) AQP4-IgG-seropositive neuromyelitis optica spectrum disorder

| **Case** | **Type of infection** | **Severity** | **Treatment** |
| --- | --- | --- | --- |
| 1 | COVID-19 infection | Non-severe | PO molnupiravia for 5 days |
|  | Bartholin abscess | Non-severe | Marsupialisation  PO co-amoxiclav for 7 days |
|  | Community-acquired pneumonia | Non-severe | PO co-amoxiclav for 7 days + PO azithromycin for 5 days |
|  | COVID-19 infection | Non-severe | PO favipiravir for 5 days |
|  | Urinary tract infection | Non-severe | PO ciprofloxacin for 7 days |
| 2 | COVID-19 infection | Non-severe | IV remdesivir for 3 days (detected during admission) |
|  | Bacterial meningitis | Non-severe | IV piperacillin/tazobactam -> IV ceftriaxone for 14 days |
|  | COVID-19 infection | Asymptomatic | PO favipiravir for 5 days |
| 3 | Urinary tract infection | Non-severe | N/A |
| 4 | Lobar pneumonia (*Haemophilus influenzae* + *Staphylococcus aureus*) | Non-severe | IV piperacillin/tazobactam -> IV levofloxacin for 14 days |
|  | Exudative tonsilitis | Non-severe | IV ceftriaxone -> PO cefdinir for 7 days |
| 5 | COVID-19 pneumonia | Non-severe | N/A |
|  | Urinary tract infection | Non-severe | N/A |
| 6 | Pulmonary tuberculosis | Asymptomatic | PO isoniazid + rifampicin + ethambutol (allergic to pyrazinamide) for 6 months |
| 7 | Urinary tract infection | Non-severe | PO ciprofloxacin for 5 days |
| 8 | Labial herpes simplex | Non-severe | PO acyclovir |
| 9 | Urinary tract infection | Non-severe | PO cefixime |
| 10* | Recurrent cholangitis (with choledochal cyst) | Non-severe | Choledochal cyst excision with hepaticojejunostomy anastomosis |
|  | Recurrent urinary tract infection | Non-severe | PO fosfomycin |
|  | COVID-19 pneumonia | Severe | Intubation  PO molnupiravir |
|  | Invasive candidaemia | Severe | IV amphotericin B -> IV micafungin -> IV fluconazole for 14 days |
| 11 | Herpes simplex | Non-severe | PO acyclovir |
| 12* | Recurrent urinary tract infection (*Escherichia coli*) | Non-severe | N/A |

Severe case was defined as requiring ICU admission.

*Patients 10 and 12 have hypogammaglobulinemia.
